# Why we should care about soft tissue interfaces when applying ultrasonic diathermy: an experimental and computer simulation study

**DOI:** 10.1186/s40349-017-0086-y

**Published:** 2017-01-27

**Authors:** Thaís Pionório Omena, Aldo José Fontes-Pereira, Rejane Medeiros Costa, Ricardo Jorge Simões, Marco Antônio von Krüger, Wagner Coelho de Albuquerque Pereira

**Affiliations:** 1 Ultrasound Laboratory, Biomedical Engineering Program/COPPE/Federal University of Rio de Janeiro - UFRJ, Rio de Janeiro, Rio de Janeiro Brazil; 2 Brazilian National Cancer Institute, INCA, Rio de Janeiro, Rio de Janeiro Brazil; 3 Centre for Informatics and Systems, University of Coimbra, Coimbra, Portugal

**Keywords:** Ultrasonic therapy, Ultrasound, Diathermy, Evidence-based practice, Practice-research gap

## Abstract

**Background:**

One goal of therapeutic ultrasound is enabling heat generation in tissue. Ultrasound application protocols typically neglect these processes of absorption and backscatter/reflection at the skin/fat, fat/muscle, and muscle/bone interfaces. The aim of this study was to investigate the heating process at interfaces close to the transducer and the bone with the aid of computer simulation and tissue-mimicking materials (phantoms).

**Methods:**

The experimental setup consists of physiotherapeutic ultrasound equipment for irradiation, two layers of soft tissue-mimicking material, and one with and one without an additional layer of bone-mimicking material. Thermocouple monitoring is used in both cases. A computational model is used with the experimental parameters in a COMSOL® software platform.

**Results:**

The experimental results show significant temperature rise (42 °C) at 10 mm depth, regardless of bone layer presence, diverging 3 °C from the simulated values. The probable causes are thermocouple and transducer heating and interface reverberations. There was no statistical difference in the experimental results with and without the cortical bone for the central thermocouple of the first interface [*t*(38) = −1.52; 95% CI = −0.85, 0.12; *p* = 14]. Temperature rise (>6 °C) close to the bone layer was lower than predicted (>21 °C), possibly because without the bone layer, thermocouples at 30 mm make contact with the water bath and convection intensifies heat loss; this factor was omitted in the simulation model.

**Conclusions:**

This work suggests that more attention should be given to soft tissue layer interfaces in ultrasound therapeutic procedures even in the absence of a close bone layer.

## Background

The first observations of interaction between ultrasound (US) and biological tissue date back to 1920. However, the useful and desirable thermal effects associated with the US radiation were recognized as a therapeutic resource in medicine only in the 1930s [1, 2].

In today’s clinical practice of physical therapy, the therapeutic application of the US has benefits such as pain reduction [3, 4], increase in collagen extensibility [5], muscle and tendon elasticity, tendon tension strength, joint amplitude, and nerve conduction velocity, and acceleration of ligament repair [6–8]. According to the literature [6, 8, 9], a therapeutic dose should be able to heat the area of interest to temperatures in the range of 40 to 45 °C for at least 5 min. This temperature depends on (a) radiation parameters, like frequency, acoustic intensity, effective radiation area, duty cycle; and (b) physical medium properties, like density, specific heat, thermal conductivity, acoustic propagation velocity, attenuation coefficient, tissue temperature, and pressure.

However, despite the substantial number of publications presenting evidence about the therapeutic effects of ultrasound, quantitative information supporting the effectiveness of the application protocols proposed for distinct clinical conditions has still not been presented.

As a result, physical therapists usually subject their patients to the US therapy on an ad hoc basis, according to general recommendations found in the equipment user’s manual. Among the most common user recommendations to avoid unwanted and painful hot spots (local intensity peaks) and overheating at the muscle/bone interface are to (a) manually move the transducer at a recommended speed (4 cm s^−1^), (b) choose a transducer displacement pattern, which can be linear (side-to-side) or circular, and (c) apply light pressure to the skin surface in the treatment area [7, 9–15].

Given the aforementioned concerns, there exists substantial cause to study the US doses, application protocols, and the resulting temperature distributions, which give rise to hot spots in the irradiated medium. This work identifies two distinct therapeutic situations, viz. the presence or absence of a bone/soft tissue interface in the propagation path of ultrasonic energy; the effect of this presence on the resulting temperature and heat distribution in the insonated tissue is also discussed.

The experimental objectives are to irradiate the phantom and then monitor the variations in temperature in a multi-layered phantom with thermocouples inserted between the layers. In the first experiment, the two layers consisted of a soft tissue-mimicking material. In the second experiment, a third layer of bone phantom was inserted. Both experimental designs were simulated by a numerical model.

## Methods

### Model simulation

The computational simulation was performed with COMSOL Multiphysics (COMSOL Multiphysics Inc., MA, USA), a commercial software that solves the acoustic wave equation coupled to the Bioheat equation [16]. Two-dimensional models were built to represent the experimental set up. The first model is composed of two layers of soft tissue-mimicking material (10 and 20 mm thick) and water. The second model has the same two layers of the soft tissue and a third layer of bone-mimicking material (1 mm thick). The transducer is placed and fixed at the surface of the first soft tissue layer. With an initial temperature of 36 °C, the US propagation takes place at 1 MHz and 1.24 W cm^−2^ for 120 s. The software generates the temperature map of both models, with absolute temperature values available.

### Experimental model

The experimental model was designed and constructed in the Ultrasound Laboratory (LUS/COPPE/UFRJ), and it consists of polyvinyl chloride (PVC) rings, temperature sensors (thermocouples), and the phantom material, similar to the system described by Omena et al. [17]^.^ Two PVC rings (internal diameter 65 mm, external diameter 75 mm), with 10-mm and 20-mm thickness, were built. They were filled with a soft tissue phantom material comprising layer 1 and layer 2 (10 and 20 mm thick, respectively).

**Fig. 1:**
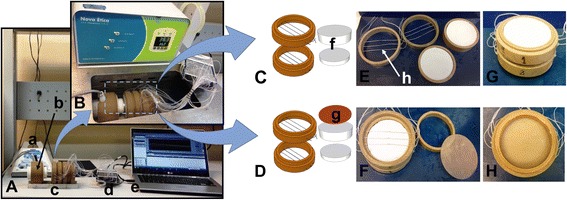
**A** Experimental setup with ultrathermostat: (*a*) ultrasound equipment, (*b*) ultrasound transducer, (*c*) calorimeter, (*d*) NI data acquisition board, and (*e*) a computer. **B** Calorimeter and ultrasound transducer immersed in the ultrathermostat water bath. **C**, **E** Calorimeter separated: thermocouple matrix, (*f*) soft tissue phantom, and (*h*) thermocouple. **D** Calorimeter separated: thermocouple matrix, soft tissue phantom, and (*g*) the cortical bone phantom of 1-mm thickness. **F** *Bottom view* of calorimeter and thermocouples before setting the synthetic cortical bone. **G** *Upper view* of calorimeter with soft tissue phantom and **H** *Bottom view* of the calorimeter after setting the synthetic cortical. **C**, **E**, and **G** are the first experimental model and **D**, **F,** and **H** are the second experimental model

Six type-K thermocouples (nickel-chromium/nickel-alumel) were built in the laboratory (0.25-mm diameter). The PVC rings were coupled (layers 1 and 2); between their interfaces, three thermocouples were positioned in parallel 10 mm apart (Fig. 1). Three other thermocouples were positioned at the end of layer 2. This is the first experimental model (Fig. 1C, E, and G). The experiment was performed entirely inside a water bath at 36 °C. The second experimental model is similar to the first one, except that a synthetic bone (Sawbones©Headquarters, WA, USA) layer of cylindrical shape, with 65-mm diameter and 1-mm thickness, was coupled to the end of layer 2 (Fig. 1D, F, and H). The model was also immersed in the same water bath at 36 °C.

All thermocouples were calibrated in a thermostat 521-2D (Nova Ética Produtos e Equipamentos Científicos Ltda., SP, Brazil) having a reference thermometer MTH 1362 W (Minipa, SP, Brazil). The measured temperature range was 20–70 °C.

The soft tissue phantom employed was made at the laboratory and was based on the standard IEC 60601-2-37 (2007) with the following materials: glycerol, water, benzalkonium chloride, silicon carbide (SiC), aluminum oxide (Al_2_O_3_ a 0.3 μm), and agar. The phantom mimics the soft-tissue average properties: propagation velocity of 1540 m s^−1^, density 1050 kg m^−3^, attenuation coefficient 0.5 dB cm^−1^ MHz^−1^, specific heat capacity 3800 J kg^−1^ K^−1^, thermal conductivity 0.58 W kg^−1^ K^−1^, thermal diffusivity 0.15 × 10^−6^ m^2^ s^−1^, conductivity 0.58 W kg^−1^ K^−1^], and thermal diffusivity 0.15 × 10^−6^ m^2^ s^−1^. The properties of the synthetic bone are attenuation coefficient 6.15 dB cm^−1^ MHz^−1^, density 1700.59 g cm^−3^, velocity 2924.31 m s^−1^, thermal conductivity 0.47 W m^−1^ °C^−1^, and specific heat 1256.34 J kg^−1^ K^−1^.

A calibration of the ultrasound equipment, Avatar III (KLD Biosistemas Equipamentos Eletrônicos Ltda., SP, Brazil), was performed. The acoustic power, radiation effective area (ERA), and beam non-uniformity ratio (BNR) measured were 4.18 W, 3.36 cm^2^, and 3.91, respectively. From these results, the effective intensity obtained is 1.24 W cm^−2^. The frequency adopted was 1 MHz, and the continuous mode and irradiation times were 120 s. The transducer was stationary.

The experimental set up included tissue models, ultrasound equipment, ultrathermostat water bath, NI data acquisition board Hi-speed USB carrier/Ni USB-9162, USB-9162 (National Instruments Corporation, TX, USA), and a computer. They were arranged as follows: the first model was immersed in the water bath at a controlled temperature of 36 °C. The ultrasound transducer was positioned in contact with the soft tissue phantom surface. The first three thermocouples were placed between the phantom layers, and the other three thermocouples were inserted into the phantom/water interface. For the second model, the cortical bone layer was introduced, and the three thermocouples were positioned between layer 2 and the bone layer.

Temperature data was obtained at layer 1/layer 2 interface (at 10 mm) and layer 2/water or layer 2/bone interface (30 mm from transducer face). Thermocouples were connected to the Hi-speed USB carrier/Ni USB-9162, which was connected to the computer by USB. Software programmed on the Labview (National Instruments Corporation, TX, USA) platform was used to register and save data. In total, 40 measurements were performed: 20 without and 20 with the compact bone. Statistical analyses, to compare heating between the two set up arrangements, were made.

### Data analysis

The number of measurements was determined based on a value of *α* = 0.05, two-tailed *t* test, expected standard deviation of residuals of 0.5, expected difference in means of 0.5, and a test power of 80%. A sample of 20 measurements per group was required to evaluate a mean difference between experiments with and without the cortical bone in the calorimeter. The Kolmogorov-Smirnov test was used to test normality; Student’s *t* test (*t*) was used to compare groups or, when appropriate, the Mann-Whitney rank sum test (*U*) was used. A significance level of *α* = 0.05 was assumed. Data analysis was carried out using Microsoft Excel® and SigmaStat Software, version 3.5 (Systat Software Inc., CA, USA).

## Results

**Fig. 2:**
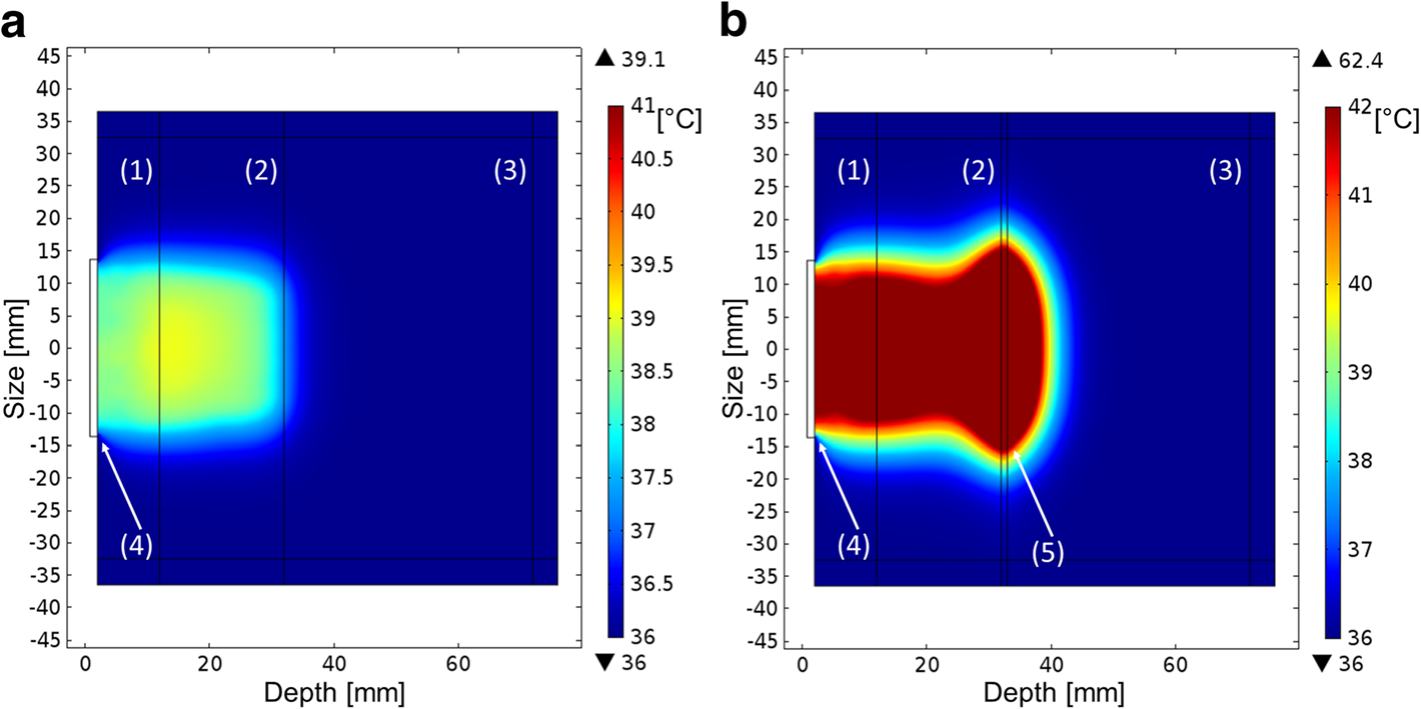
**a** Thermal pattern in the calorimeter without the cortical bone. **b** Thermal pattern in the calorimeter with the cortical bone. The *number* (*1*) is the tissue phantom—10 mm, (*2*) is the tissue phantom—20 mm, (*3*) water, (*4*) transducer membrane, and (*5*) bone phantom

The experiments with the calorimeter simulated in COMSOL, and the resulting temperature maps are shown in Fig. 2.

The maximum temperature on each thermocouple at 10 and 30 mm, obtained by theoretical simulations and experimental results, are shown in Table 1.

**Table 1:** Temperature on thermocouples at 10 and 30 mm obtained by theoretical simulations and experimental (40 experiments with and without the cortical bone)

		Simulation [°C]		Experimental [°C]	
		Without the bone^a^	With the bone^a^	Without the bone^b^	With the bone^b^
10 mm	TP1	38.57	42.59	37.52 (0.69)	38.66 (0.96)
	TP2^c^	39.04	43.59	42.01 (0.47)	42.37 (0.96)
	TP3	38.58	42.60	37.94 (0.47)	38.47 (0.80)
30 mm	TP4	37.07	53.75	36.31 (0.16)	37.42 (0.76)
	TP5^c^	37.34	61.15	36.70 (0.21)	39.23 (1.01)
	TP6	37.08	53.74	36.28 (0.25)	37.21 (0.66)

TPi → i-th Thermocouple (i = 1 to 6)

^a^Values are mean

^b^Values are mean (SD)

^c^Central thermocouples

Comparison between the experimental results with and without the cortical bone shows that all thermocouples presented statistical differences: 1 [*U* = 320.00; *p* = 0.001], 3 [*U* = 71.00; *p* ≤ 0.001], 4 [*U* = 378.00; *p* ≤ 0.001], 5 [*U* = 400.00; *p* ≤ 0.001], and 6 [*U* = 377.00; *p* ≤ 0.001]; except thermocouple 2: [*t*(38) = −1.52; 95% CI = −0.85, 0.12; *p* = 0.14].

## Discussion

From the simulation results (Fig. 2), we can see that the heating covers a broader area and reaches higher temperatures when the bone is present. The main difference regarding wave propagation is reverberation between the bone surface and the face of the transducer. We have made a brief calculation of the percentage of the energy that is “imprisoned” in-between the transducer—bone surfaces and it is in the range of 12 to 45%, for incident angles between 0 and 15°. We have considered the longitudinal and shear waves. Standing waves happen in a more specific scenario, but reverberation will always happen.

It is widely accepted that ultrasonic irradiation at 1 MHz produces deep heating in tissues (from 30 to 50 mm) [9–11, 14]. This work suggests that significant heat buildup occurs in more superficial areas. Therefore, more attention should be given in the administration of the US therapy when subjecting a patient to this kind of irradiation.

This remark is supported by the results of the present simulation, where the heating in two different multi-layer phantom configurations described above, were studied.

Simulations indicated that, in the first case, the phantom constructed with two soft tissue layers had heat variations as a function of the ultrasonic field intensity and decreases in heat according to changes in attenuation with change in distance from the source. A buildup effect, which can be attributed to backscattering and intensity distribution in the near field, was observed. Simulation results for the model, without bone layer, show a heated region at therapeutic level (above 39 °C) between 5 and 25 mm depth (the first interface included), with the rest of the phantom below this temperature.

In the second case of the simulation, set up was modified by adding a third bone layer at the end of the second soft-tissue layer. When the bone layer is included, there is a general rise in temperature, especially at the soft tissue/bone interface. This is basically due to the amount of energy that is reflected back into the soft tissue layers. All the phantom layers are above 42 °C, the heated area is laterally spread, and there are dangerous hotspots around the bone interface (thermocouples 4, 5, 6, Table 1). As there is mode conversion in the wave propagation in the bone, the absorption mechanisms are stronger (shear and pressure waves are present). Nevertheless, the temperature of the first interface increased only about 4 °C (thermocouples 1, 2, 3, Table 1).

Actual experimental results are in agreement with simulated results: the first interface has higher temperatures than the second interface, when there is no bone layer. When the bone layer is introduced, the first and the second interfaces have increased temperatures and the higher temperatures are in thermocouples 2 and 5, as in the simulation. However, the temperature increase was different in the two cases: in the simulation, there was an increase on the first interface of about 4 °C and on the second interface of 16 to 23 °C. In the experimental case, the increase was lower about 1 °C on the first interface and 1 to 3 °C on the second interface. This can be due to experimental limitations. For example, the interfaces between layers are not bonded together, they have a thin water layer with the thermocouples inserted, causing propagation phenomena to differ from the simulated models.

For the experimental set up, even though the increase in the temperature average was low, statistical analysis indicates differences between the cases (with and without the bone) with the exception of thermocouple 2.

The thermocouple dimension is negligible (about 1/6 of the wavelength for 1 MHz) and it is inside a thin water layer in the experimental case, so we assumed that viscous heating is negligible. The simulation used the same application time of the experimental case, and it was not the equilibrium temperature. Usually, in clinical therapy it is used 300 s moving the transducer, but with the transducer fixed, the irradiation time selected was 120 s, which is the equivalent time on a surface during therapy (1 to 2 min in each position, in average). So, the US therapeutic equipment was turned off on the selected time, before reaching the equilibrium temperature.

Thermal and nonthermal effects may occur simultaneously. As an intensity of 1.24 W cm^−2^ was used at this intensity, only stable cavitation may exist, while transient cavitation, which seems to present a more important contribution to heating, is not happening yet.

Limitations of the present study are (a) the stationary position of the experimental transducer versus consistent movement of it in practical application and treatment, (b) shorter irradiation time (120 s) used compared to actual case averages, (c) blood perfusion was not included, which should result in a reduction in the absolute temperature values, and (d) a small number of temperature sensors were employed.

Future research may be performed by applying phantoms of the skin, fat, muscle, and bone, with more thermocouples and perfusion will be incorporated in the simulated model.

## Conclusions

This work presented a theoretical and experimental verification of the general temperature increase caused by the presence of a bone layer in a common treatment procedure of physio-therapeutic ultrasound. This temperature increase happens not only in the muscle/bone interface vicinity but also in the soft tissue interfaces that are more superficial, so a broader region is affected. Another important result is that the interface temperature between soft tissues experiences a significant increase during the therapeutic application, even in the absence of a bone layer. To date, we have no knowledge of reported, similar experimental or simulated results. These conclusions substantiate precautionary measures needed in the application of the US therapy by practitioners, whether there is presence or absence of the bone.

## Abbreviations

BNR: Beam non-uniformity ratio
ERA: Radiation effective area
PVC: Polyvinyl chloride
US: Ultrasound
